# Toll-like receptor 4 plays a vital role in irritable bowel syndrome: a scoping review

**DOI:** 10.3389/fimmu.2024.1490653

**Published:** 2024-12-19

**Authors:** Xuemeng Wan, Liyuan Wang, Zhiling Wang, Chaomin Wan

**Affiliations:** ^1^ Department of Pediatrics, West China Second University Hospital of Sichuan University, Chengdu, China; ^2^ Key Laboratory of Birth Defects and Related Diseases of Women and Children, Ministry of Education, Chengdu, China; ^3^ National Health Commission Key Laboratory of Chronobiology, Sichuan University, Chengdu, China

**Keywords:** irritable bowel syndrome, toll-like receptor 4, inflammation, visceral hypersensitivity, treatment

## Abstract

**Background:**

Irritable bowel syndrome (IBS) is a common gastrointestinal disease. Recently, an increasing number of studies have shown that Toll-like receptor 4 (TLR4), widely distributed on the surface of a variety of epithelial cells (ECs) and immune sentinel cells in the gut, plays a vital role in developing IBS.

**Objectives:**

We sought to synthesize the existing literature on TLR4 in IBS and inform further study.

**Methods:**

We conducted a systematic search of the PubMed, Embase (Ovid), Scopus, Web of Science, MEDLINE, and Cochrane Library databases on June 8, 2024, and screened relevant literature. Critical information was extracted, including clinical significance, relevant molecular mechanisms, and therapeutic approaches targeting TLR4 and its pathways.

**Results:**

Clinical data showed that aberrant TLR4 expression is associated with clinical manifestations such as pain and diarrhea in IBS. Aberrant expression of TLR4 is involved in pathological processes such as intestinal inflammation, barrier damage, visceral sensitization, and dysbiosis, which may be related to TLR4, NF-κB, pro-inflammatory effects, and CRF. Several studies have shown that many promising therapeutic options (i.e., acupuncture, herbs, probiotics, hormones, etc.) have been able to improve intestinal inflammation, visceral sensitization, intestinal barrier function, intestinal flora, defecation abnormalities, and depression by inhibiting TLR4 expression and related pathways.

**Conclusion:**

TLR4 plays a crucial role in the development of IBS. Many promising therapeutic approaches alleviate IBS through TLR4 and its pathways. Strategies for targeting TLR4 in the future may provide new ideas for treating IBS.

## 1 Introduction

Irritable bowel syndrome (IBS), a functional gastrointestinal disease, is characterized by recurring abdominal pain and alterations in stool frequency or shape (1). Using Bristol stool grade, IBS patients are classified based on their abnormal defecation patterns into diarrhea-predominant IBS (IBS-D), constipation-predominant IBS (IBS-C), mixed IBS (IBS-M) and unclassified IBS (2). Globally, the incidence of IBS varies, approximately 10.1% (9.8%-10.5%) using the Rome III criteria and 3.8% (3.6%-4.0%) using the Rome IV criteria (3). Although IBS is not associated with increased mortality (4), it significantly impacts health-related quality of life, social functioning and psychosocial factors (5–8). Moreover, it imposes a substantial social and economic burden (1, 9, 10). Most costs incurred by IBS patients are due to productivity loss, and direct healthcare expenses are driven by IBS-related comorbidities (11). However, its underlying pathophysiological mechanisms are not fully understood, possibly involving factors like gut-brain axis dysfunction, stress, visceral hypersensitivity (VHS), altered gut motility, barrier function destruction, gut microbiome disorders, intestinal inflammation, immune activation, genetic factors, etc. (1). And as a consequence, no medical therapy is proven to alter the natural history of IBS, usually focus on alleviating symptoms. Therefore, new targets for IBS prevention and treatment have become important. In recent years, increasing numbers of studies have demonstrated that the Toll-like receptor (TLR) 4 plays a pivotal role in developing IBS (12, 13).

TLRs, members of the transmembrane pattern recognition receptor family, play an essential role in innate immune responses and bridge innate and acquired immunity. TLRs are involved in mucosal immune response, barrier function, cell adhesion, cell proliferation and migration, protection from pathogens, repair of epithelial cell injury, etc. (14). Thus, dysregulation of the TLRs signaling pathway contributes to the development and progression of various diseases such as autoimmune diseases, cancer, infections and chronic inflammation (15). Almost all TLRs are expressed in the small intestine and colon intestinal epithelial cells (ECs) (16).

TLR4 is one of the earliest transmembrane pattern recognition receptor family members to be studied. TLR4 is expressed in humans and mice’s colon and ileum crypts (17). TLR4 signaling is essential for maintaining intestinal homeostasis, and its hyperactivation is a crucial driver of many disease states affecting gastrointestinal function (18). Early life stress affects susceptibility to IBS by modulating TLR4 (19). One study found that TLR4 mRNA expression was associated with the intensity of abdominal pain in IBS-D patients (20). TLR4 can promote the up-regulation of pro-inflammatory cytokines by activating the transcription factors nuclear factor-kappa B (NF-κB) via the adaptor protein myeloid differentiation primary response gene 88 (MyD88), ultimately affecting the intestinal barrier, VSH (13, 21). In addition, increased intestinal permeability in IBS patients promotes the activation of TLR-dependent immune responses.

There are no relevant review articles on the role of TLR4 in the pathological mechanisms of IBS. We have collected the current essential studies on the expression of TLR4 in IBS, the mechanism of action, and the drugs targeting TLR4 for the treatment of IBS through a scoping review, which may be instructive for a complete understanding of the biological function of TLR4 in the development of IBS.

## 2 Method

### 2.1 Information sources and search strategies

Six common life-science databases (PubMed, EMBASE, Scopus, Web of Science, MEDLINE, and Cochrane Library) were applied to identify those studies that met the review criteria. In the PubMed database, we searched the eligible studies by using the following keywords: (((((((Toll-Like Receptor 4 [MeSH Terms]) OR (TLR4 [Title/Abstract])) OR (TLR-4 [Title/Abstract])) OR (Toll Like Receptor 4 [Title/Abstract])) OR (Toll-4 Receptor [Title/Abstract])) OR (Toll 4 Receptor [Title/Abstract])) OR (TLR4 Receptor[Title/Abstract])) OR (Receptor, TLR4[Title/Abstract]) AND ((((((irritable bowel syndrome [MeSH Terms])) OR (Irritable Bowel Syndromes [Title/Abstract])) OR (Syndrome, Irritable Bowel [Title/Abstract])) OR (Syndromes, Irritable Bowel [Title/Abstract])) OR (Colon, Irritable [Title/Abstract])) OR (Irritable Colon [Title/Abstract]). Reference lists of relevant publications were searched to identify more relevant studies. There were no restrictions on the language and date of publication. The literature was last searched on 7 June 2023.

### 2.2 Inclusion criteria

The study reports the expression of the TLR4 gene in irritable bowel syndrome compared to healthy controls, the pathogenesis involved in TLR4 in IBS, and therapeutic approaches targeting TLR4 for the treatment of IBS are included in the study. Study subjects included humans, rat rats, mouse mice, and cell cells. There are no language or publication status restrictions. Meetings and lack of full-text literature were excluded.

### 2.3 Literature selection

Study selection occurred in three stages: First, duplicate publications were immediately eliminated. Second, two researchers independently reviewed titles and abstracts of the literature, and literature that did not meet the inclusion or exclusion criteria was discarded. Articles whose abstracts needed to provide more information to determine whether they were excluded were included directly in the full-text review stage. Third, full-text reviews were conducted independently by two researchers. Disagreements throughout the process were resolved by discussion or input from a third reviewer if required.

### 2.4 Data extraction

Two researchers worked together independently to extract data from articles that fit the topic of this study and then exchanged them for validation. A third researcher resolved the differences. Variables included clinical sample size, diagnostic criteria, study object, disease modelling method, the status of TLR4, effect on IBS, associated genes or pathways, and the main findings of the independent study.

## 3 Result

**Figure 1:**
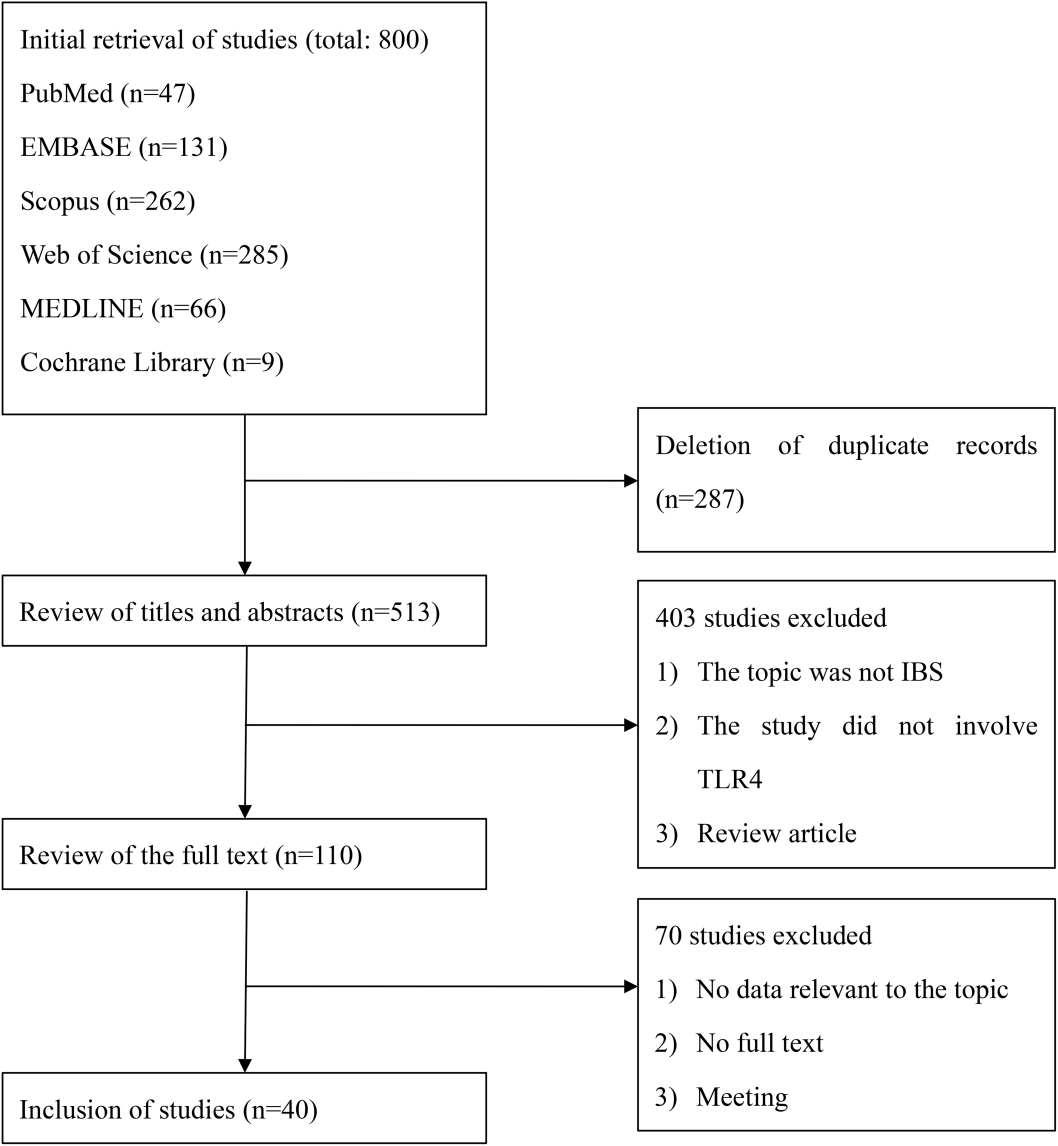
Study selection.

Six database searches identified 800 citations. After removing duplicates, 513 unique citations were screened for titles and abstracts. Of these citations, 110 met the criteria for full-text review. We excluded 70 studies because they did not have relevant data, could not be found in full text, contained insufficient information to assess the relationship between IBS and TLR4, or were conference literature. Finally, 40 studies were included. This whole process is outlined in **Figure 1**.

**Figure 2:**
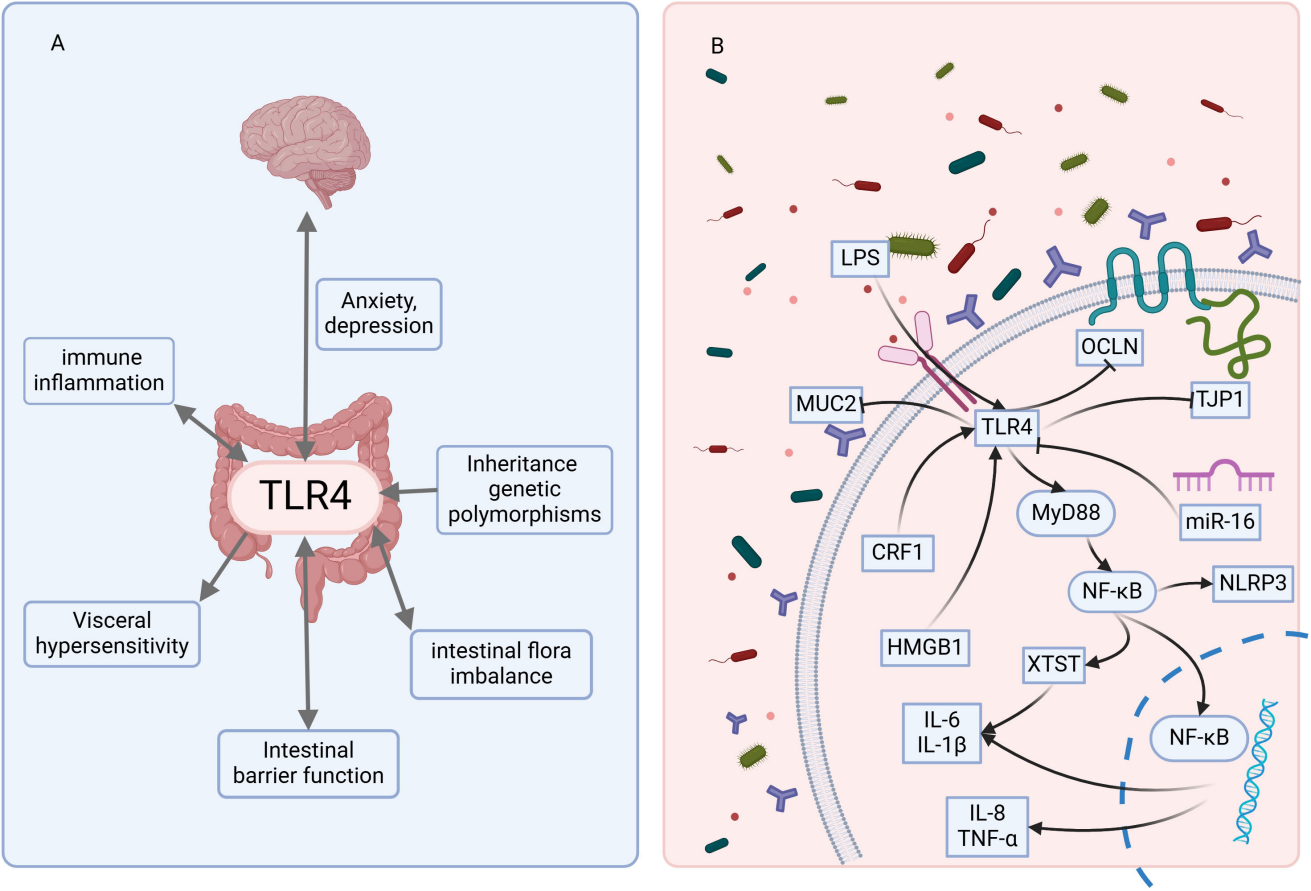
The mechanisms and pathways of TLR4 involvement in IBS. **(A)** indicates that TLR4 is involved in multiple pathogenic mechanisms of IBS. **(B)** indicates that TLR4 is involved in the molecular pathways involved in developing IBS. LPS, lipopolysaccharide; MUC2, Mucin 2; TJP1, Tight junction protein 1; OCLN, Occludin; HMGB1, High mobility group box 1; CRF1, Corticotropin-releasing factor receptor subtype 1. The figure was created using BioRender mapping software (https://BioRender.com).

Based on the 40 included studies, we summarized the mechanisms and pathways of TLR4 involvement in IBS (**Figure 2**).

A total of 13 clinical research articles describing the expression of TLR4 in patients with IBS were identified in the literature search; 11 (11/13, 84.62%) articles showed that the expression of TLR4 was up-regulated in IBS compared with health control (HC), and only 2 (2/13, 15.38%) of them suggested that no change in TLR4 was found in IBS (**Table 1**). In addition to changes in TLR4 expression, the expression of TLR2, TLR5, TLR9, and inflammatory factors (interleukin (IL)-1α, IL-1β, IL-6 and IL-8) were up-regulated and anti-inflammatory factor (IL-10) was down-regulated (**Table 1**).

**Table 1:** Studies reported the clinical findings the clinical significance of TLR4 in IBS.

Study	Publication year	Clinical sample size	Diagnostic criteria	Status of TLR4	Associated genes or pathways	Main findings
Belmonte et al. (22)	2012	48 IBS patients (37 F, mean age 49 ± 14 years) and 31 HCs (17 F, mean age 57 ± 14 years)	Rome III criteria	Activated	Increased TLR2, IL-8, and IL-1β, impaired PPARγ	Increased expression of TLR4 and TLR2 in the descending colon was observed in IBS-M patients alone, neither IBS-D nor IBS-C, resulting in enhanced expression of mucosal proinflammatory cytokines and impaired PPARγ expression.
Brint et al. (23)	2011	26 IBS patients (all F, mean age 37.9 years) and 19 HCs (all F)	Rome II criteria	Activated	Increased TLR5	The expression of TLR4 and TLR5 in IBS patient’s colon mucosal showed up-regulation.
Compare et al. (24)	2017	10 post-infectious IBS-D (PI-IBS D) patients (4 F, mean age 52 years), 10 HCs (5 F, mean age 48 years)	Rome III criteria	Activated	Increased IL-1α, IL-6, and IL-8, decreased IL-10	TLR-4 protein expression was significantly higher in PI-IBS D patients than HC in both ileal and colonic mucosa.
Dlugosz et al. (25)	2017	22 IBS patients (17 F, median age, 39 [18-66] years), 14 HCs (12 F, median age, 42 [22-61] years)	Rome II criteria	Activated	Increased TLR5 and TLR9	The higher mRNA expression of TLR4, TLR5, and TLR9 in jejunum mucosa suggests the involvement of bacteria or dysregulation of the immune response to commensal flora in small bowel mucosa in IBS patients.
Guo et al. (26)	2016	20 IBS-D patients (13 F, mean age 42.9 ± 12.5 years), 20 HCs (15 F, mean age 41.1 ± 11.5 years)	Rome III criteria	Activated	Increased TLR2	Increased expression of TLR4 and TLR2 in the IBS-D’s descending colon mucosal may be related to Bacteroidetes and Clostridium.
Jalanka et al. (27)	2021	43 IBS-D patients (26 F, mean age 40.85 [19-69] years) and 21 HCs (16 F, mean age 42.8 [20-75] years)	Rome III criteria	Activated	Increased Myd88	Elevated TLR4 in IBS-D sigmoid colon tissue significantly correlated with MYD88.
Koçak et al. (28)	2016	51 IBS patients (43 F, mean age 38.90 ± 10.42 years) and 15 HCs (11 F, mean age 37.90 ± 10.47 years)	Rome III criteria	Activated	Increased TLR2, NO	The colonic tissue levels of TLR4 in the IBS-D and IBS-C subgroups were significantly higher, and IBS had immune dysregulation and oxidative stress.
Linsalata et al. (29)	2018	39 IBS-D patients (33 F, mean age 39.7 ± 7.2 years) and 20 HCs (13 F, mean age 40.05 ± 12.2 years)	Rome III criteria	unchanged	Increased IL-6 and IL-8, LPS not changed	There was no significant difference in the concentrations of LPS and TLR-4 in the plasma of IBS-D patients compared to the control group.
Lobo et al. (20)	2017	25 IBS-D patients (17 F, mean age 37.4 ± 2.1 years) and 16 HCs (7 F, mean age 32.1 ± 2.3 years)	Rome II criteria	Activated	TLR2 has no significant difference	TLR4 is upregulated in the jejunal mucosa of IBS-D patients, and its expression is correlated with the intensity of abdominal pain in these patients.
Shukla et al. (30)	2018	47 IBS patients (8 F, median age,34 [19-68] years) and 25 HCs (3 F, median age, 40 [20-68] years)	Rome III criteria	Activated	Increased CXCL-11 and CXCR-3, TLR2 not changed	TLR4 was upregulated in IBS patients, particularly in patients with IBS-D, and there was a positive correlation between TLR4 mRNA levels and weekly stool frequency in IBS patients.
Jizhong et al. (31)	2016	102 IBS patients (39 F) and 102 HCs (45 F), age-matched	Rome III criteria	Activated	Increased TLR2, CRF1, CRF2, IL-6, decreased IL-10	TLR4 and TLR2 were upregulated in peripheral blood samples of IBS patients, especially in IBS patients with depression.
Wang et al. (32)	2008	30 IBS-D patients (16 F, mean age 47 ± 14 years) and 12 HCs (7 F, mean age 4l ± 4 years)	Rome III criteria	Activated	Increased NF-κB	TLR4/NF-κ B signaling pathway was activated in the intestinal mucosa of IBS-D patients.
Yoshimoto et al. (33)	2022	31 IBS patients (17 F, mean age 47.0 ± 18.2 years) and 31 HCs (18 F, mean age 58.0 ± 12.0 years)	Rome III criteria	unchanged	Increased CXCL-11 in the duodenum, TLR5, CXCR-3, and IL-6 not changed	There was no significant difference in the expression levels of TLR4 in the duodenum, ileum, cecum, and rectum.

The fields in parentheses in this column for the study object refer to gender and age. The F represents females. PPARγ, Peroxisome proliferator-activated receptor-γ; NO, Nitric oxide; CXCL-11, C-X-C motif chemokine ligand 11; CXCR-3, C-X-C motif chemokine receptor 3.

Ten studies reported the molecular mechanisms underlying the role of TLR4 in IBS (**Table 2**). The development of IBS disease was found to involve the TLR4/MyD88/NF-κB signaling pathway primarily. Activation of TLR4 and its signaling pathway can contribute to IBS development by promoting inflammation and mediating visceral sensitization and stool abnormalities (**Table 2**). MicroRNA was involved in IBS; research indicated that miR-16 inhibited the TLR4/NF-κB/X-inactive specific transcript (XIST) axis to relieve IBS-D (13).

**Table 2:** Studies reported the molecular mechanisms underlying TLR4-mediated IBS.

Study	Study object	Disease modeling method	Status of TLR4	Effect on IBS	Associated genes or pathways	Main findings
Bao et al. (34)	Wistar rats (F)	Acute and chronic stress (3 weeks)	Activated	Promoted inflammation	Activated the expression of MyD88, NF-κB, IL-1β, IL-6	IBS-D may increase inflammatory factors through activating the TLR4/MyD88/NF-κB signaling pathway, resulting in disease progression.
Chen et al. (35)	SD rats (M and F)	Neonatal colorectal distension (CRD)	Activated	Promoted inflammation, mediated VHS	TLR4/MyD88/NF-κB pathway, IL-1β and TNF-α	Neonatal colonic irritation (CI) stimulated the production of IL-1β and TNF-α through the TLR4/MyD88/NF-κB signaling pathway in the spinal cord, contributing to the VHS response induced by neonatal CI in rats.
He et al. (36)	Wistar rats (F)	Acute and chronic stresses (4 weeks)	Activated	Promoted inflammation	Upregulated NF-κB (p65), MyD88, IL-8, TNF-α, downregulated IL-10	TLR4/NF-κB signal pathway played an essential role in the modulation of inflammatory responses in IBS-D.
Rodríguez-Palma et al. (37)	Swiss webster mice (M and F)	Chronic restraint stress	Activated	Promoted VHS, anxiety-like behaviors	HMGB1	Highlight the significant role of the HMGB1/TLR4 pathway in chronic stress-induced VHS, anxiety-like behaviors.
Wu et al. (38)	SD rats (M)	Neonatal rats underwent CRD	Activated	Promoted VHS	NF-κB, Pellino-1	The downregulation of Pellino-1 in the fastigial nucleus acted through the TLR4/NF-κB pathway to protect against chronic VSH in a CRD rat model.
*Xi et al. (13)	NCM460 cell/NIH mice (both genders equally divided)	LPS 2ug/ml/acetic acid instillation	Activated	Inhibited enterocyte viability, promoted apoptosis and inflammation, decreased tight junction integrity/shortened defecation intervals, raised water content in stool, promoted VHS	miR-16, XIST, IL-1β, and IL-6	*In vitro* and *in vivo* experiments have validated that miR-16 inhibited the TLR4/NF-κB/XIST axis to relieve IBS-D.
Xu et al. (39)	SD rats	Neonatal maternal separation (NMS) and Water avoidance stress (WAS)	Activated	Promoted VHS	OT receptor, TLR4/MyD88/NF-κB, TNF-α and IL-1β	Exogenous OT attenuated stress-induced VHS and Enteric glial cell activation, mediated by TLR4/MyD88/NF-κB signaling.
Yuan et al. (40)	SD rats (M)	10-d-old pups acetic acid instillation	Activated	Promoted VHS	NF-κB, CBS	Activation of TLR4 by neonatal CI upregulated CBS expression, which is mediated by the NF-κB signaling pathway, thus contributing to VHS.
Zhang et al. (41)	SD rats (M)	Neonatal CRD	Activated	Promoted VHS	MyD88/NF-κB signaling, TNF-α and IL-1β	Activation of CRF neurons and microglia in the hypothalamic paraventricular nucleus participates in the pathogenesis of VHS through TLR4/MyD88/NF-κB signaling.
Tang et al. (12)	C57BL/10JNju (Tlr4) and C57BL10/ScNJNju (Tlr4) mice	NMS	Activated	Promoted VHS	MyD88, IL-1β and TNF-α, CRF, CRF1	NMS-induced VHS is associated with increased TLR4 signaling in the PVN.

The fields in parentheses in this column for the study object refer to gender. F represents females, and M represents males. * The literature includes both cell and animal experiments, separated by a slash, with the results of the cell experiments before the slash and the results of the animal experiments after the slash. OT, Oxytocin; CBS, Cystathionine beta synthetase; CRF, Corticotropin-releasing factor; PVN, Paraventricular nucleus.

We searched 18 studies that provided the data of specific treatments-targeted TLR4 for IBS (**Table 3**). Treatments included herbal medicine, moxibustion, electroacupuncture (EA), probiotics and its postbiotic (PB) element, diet, hormones, etc. (**Table 3**). These treatments decreased inflammation, attenuated VHS and depressive-like behavior, and improved abnormal defecation and intestinal flora through TLR4/MyD88/NF-κB signaling pathway and TLR4/NF-κB/NLR family pyrin domain-containing protein 3 (NLRP3) pathway (**Table 3**).

**Table 3:** Studies reported the treatments targeted by TLR4 in IBS.

Study	Study object	Disease modeling method	Intervention or treatment	Status of TLR4	Effect on IBS	Associated genes or pathways	Main findings
Arie et al. (42)	SD rats (M)	WAS	Grape seed extract (GSE)	Inhibited	Decreased VHS	NA	GSE ameliorated repeated WAS-induced visceral allodynia and colonic hyperpermeability via inhibition of TLR4.
Chimienti et al. (43)	Newborn Wistar rats	NMS	Ketogenic Diet (KD)	Inhibited	Inhibited inflammation	COX-2, SOD1, SOD2, Beclin-1 and LC3 II	Feeding animals with KD elicited compensatory mechanisms that reduced inflammation and oxidative stress, restored mitochondrial function, and restored baseline autophagy.
Chu et al. (44)	SD rats (M)	Chronic restraint combined with gavage of Senna-leaf solution	Moxibustion at “Tianshu” (ST25) and “Shangjuxu” (ST37)	Inhibited	Improved diarrhea symptoms and VHS, alleviated inflammation	TLR4/MyD88/NF-κB signaling pathway	Moxibustion may alleviate IBS-D symptoms by inhibiting the TLR4/MyD88/NF-κB signaling pathway and reducing the expression of inflammatory factors.
Compare et al. (24)	10 PI-IBS D patients (4 F, mean age 52 years),10 HC (5 F, mean age 48 years)	Ex-vivo organ culture model	Lactobacillus casei DG (LC-DG) and its PB	Inhibited	Attenuated mucosal inflammatory response	IL-1α, IL-6, IL-8, IL-10	LC-DG and its PB attenuated the inflammatory mucosal response in an ex-vivo organ culture model of PI-IBS D.
Ding et al. (45)	SD rats (M)	Acetic acid enema plus restraint stress	Wumei Pill	Inhibited	Improved abnormal bowel movements and VHS	TLR9, NF-κB	Wumei Pill had a significant therapeutic effect on IBS-D by restraining the transmission of the TLR4/9-NF-kB signaling pathway.
Elbadawi, M et al. (46)	Mouse intestinal organoid	Exposure to CRF	STW 5-II	Inhibited	Reduced inflammation	TLR4/MyD88/NF-κB	STW 5-II may alleviate inflammation through inhibited TLR4/MyD88/NF-κB pathway.
Huang et al. (47)	rats	NMS combined with *Campylobacter jejuni* (ATCC81-176) treatment	QingHuaZhiXie prescription	Inhibited	Reduced diarrhea index and intestinal hypersensitivity	TLR4/MyD88/NF-κB pathway	QingHuaZhiXie prescription reversed the imbalance of cytokines by acting on the TLR4/MyD88/NF-κB pathway, contributing to the release of IBS-D symptoms.
Ruiz-Malagón et al. (48)	SD rats (M)	Deoxycholic acid enema	Serpylli herba extract (SHE)	Inhibited	Improved VHS and immune status	NA	SHE may reduce IBS mucosal inflammation by inhibiting TLR4.
Lin et al. (49)	ICR mice (M)	Restraint stress (10h)	Melatonin (MT)	Inhibited	Mitigated colonic microbiota dysbiosis and intestinal inflammation	NF-κB	MT may mitigate “restraint stress”-induced colonic microbiota dysbiosis and intestinal inflammation by inhibiting the activation of the TLR4/NF-κB pathway.
Liu et al. (50)	Wistar rats (M)	Repeat WAS	Saccharomyces boulardii (Sb)	Inhibited	Improved colonic hypermotility and decreased inflammation	Inhibited IL-6, IL-1β, and IFN-γ, increased IL-10	Sb improved repeated WAS-induced colonic hypermotility and decreased TLR4 expression in the colon.
Maqoud et al. (51)	Caco-2 cells	LPS	Lens culinaris Medik extract (LE)	Inhibited	Reduced inflammation	Inhibited NF-κB	LE suppressed TLR4 and NF-κB and inhibited inflammation.
Mylene et al. (52)	C57/Bl6 mice (M)	WAS	Probiotic combination Lactibiane Tolerance (LT)	Inhibited	Prevented epithelial barrier impairment	NA	LT prevention of WAS-induced epithelial barrier integrity loss could be linked to this treatment’s ability to decrease WAS-induced TLR4 up-regulation in the colonic mucosa.
Xia et al. (53)	SD rats (M)	WAS	Apigenin	Inhibited	Mitigated VHS and colonic hypermotility	TLR4/MyD88/NF-κB pathway	Apigenin inhibited Mast cells (MCs) activation through the TLR4/Myd88/NF-κB pathway, which improved visceral sensitivity.
Zhang et al. (54)	SD rats	Restraining stress and acetic acid enema	Sancao Lichang Decoction	Inhibited	Improved abnormal defecation, inhibited the inflammatory response, and improved intestinal mucosal barrier function	TLR4/MyD88/NF-κB	Sancao Lichang decoction may reduce IBS-D by inhibiting the TLR4/MyD88/NF-κB pathway.
Zhang et al. (55)	SD rats (M)	NMS in combination with restraint stress and gavage of senna decoction	Chang-Kang-Fang (CKF)	Inhibited	Attenuated VHS, reduced the number of fecal pellets, improved inflammatory response and colonic barrier function	TLR4/NF-κB/NLRP3 pathway	CKF might mitigate the symptoms of IBS-D rats by inhibiting the TLR4/NF-κB/NLRP3 pathway.
Zhu et al. (56)	Wistar rats (both genders at a ratio of 1:1)	Gavage of senna decoction plus restraint stress	Berberis heteropoda Schrenk roots (BHS)	Inhibited	Reduced VHS, improved abnormal defecation and intestinal flora	NF-κB, TLR2	BHS downregulated the mRNA expression of TLR2, TLR4, and NF-κB in brain and colon tissues, improving IBS-D.
Zhu et al. (57)	SD rats (M)	Chronic restraint stress	Xiaoyaosan (XYS)	Inhibited	Improved depressive-like behavior and weight loss	TLR4, MyD88, NF-κB, NLRP3	XYS can improve depressive-like behavior in rats by suppressing the activation of the TLR4/NLRP3 inflammasome signaling pathway.
Yang et al. (58)	SD rats (newborn M)	Acetic acid instillation	EA	Inhibited	Decreased VSH	IL-8, IL-1β	The potential mechanism underlying the reduction in visceral hypersensitivity through the stimulation of Zusanli using EA involves suppressing TLR4 expression in the MCs of colonic tissues.

The fields in parentheses in this column for the study object refer to gender and age. F represents females, and M represents males. COX-2, cyclooxygenase; SOD, superoxide dismutase; NA, Not available.

## 4 Discussion

By summarizing the literature on the progress of TLR4 in IBS disease research, it is clear that TLR4 plays a vital role in developing IBS. TLR4 is involved in the pathogenesis of IBS, including low-grade inflammation, increased visceral sensitivities, intestinal barrier damage, intestinal flora dysbiosis, defecation abnormalities, etc.

TLR4 expression was upregulated in IBS patients (20, 22–28, 30–32). However, the results of TLR4 expression in different subtypes were inconsistent. Belmonte et al. reported significant differences by subtype with a 2-fold increase in IBS-M, more significant than in IBS-D (22). While Shukla et al. found the most significant increase in IBS-D (30). Five IBS-D-only publications, including one PI-IBS D, showed upregulation of TLR4 (20, 24, 26, 27, 32). Only one article on IBS-D patients suggests no change in TLR4 (29).

In two clinical studies of IBS that showed no significant change in TLR4 (29, 33), the disease was diagnosed using the Rome III diagnostic criteria. One specimen was peripheral blood serum, and the other study took mucosal tissues from multiple sites in the gut. Overall, there were no significant differences from the other studies in terms of IBS diagnostic criteria, IBS subtypes, sample types, or anatomic locations. Analyze why the results of these two studies are inconsistent with those of other studies. Yoshimoto et al.’s study had the presence of taking anti-flatulent and antidepressant drugs, which may have influenced the results. Disease heterogeneity is a primary reason for inconsistent results, such as concomitant symptom involvement, with one study showing that TLR4 expression was higher in IBS patients with concomitant depression (31). In addition, the sample sizes in these pieces of literature are small, which can lead to excessive random errors in the results. Future large-scale studies are needed to investigate the expression pattern of TLR4 in IBS, whether there are differences in expression between subtypes and the effect of concomitant symptoms such as anxiety and depression on TLR4 expression in IBS. This will help us to understand the disease, the relationship between the different subtypes, and the management of the disease. Other factors that may have influenced the results were genetic, environmental, etc.

### 4.1 TLR4 and intestinal immune and inflammatory activation

Low-grade mucosal inflammation and immune dysfunction are some of the main pathogenic mechanisms of IBS. Several clinical studies have found elevated levels of pro-inflammatory cytokines (such as IL-1α, IL-1β, IL-6, IL-8, IL-17, TNF-α, CXCL-11 and CXCR-3)and reduced levels of the anti-inflammatory cytokine IL-10 in patients with IBS (22, 24, 29–31, 59–62). There was a correlation between inflammatory factors and IBS symptoms and quality of life (60). Meanwhile, mRNA levels of TLR-4 in IBS patients were positively correlated with the inflammatory factor IL-6 (30).Activation of TLR4 induces the expression of IL-1, IL-6 and IL-8 (63). NF-κB, which TLR4 can activate, is a central mediator in the induction of pro-inflammatory genes and plays a role in both innate and adaptive immune cells (64). Three studies showed that inflammatory factor production may be caused by the TLR4/MyD88/NF-κB pathway in an animal model of IBS-D, which promotes the development of IBS-D (34–36). Inflammatory factor (IL-6, IL-1β) expression was found to be attenuated by inhibition of the TLR4/NF-κB/XIST pathway in LPS induced damage to human normal colonic ECs (13). Furthermore, Belmonte et al. found that the imbalance between elevated levels of TLR4 and the impaired expression of PPARγ, a potential inhibitor of colonic inflammation, suggests an altered response to luminal bacteria leading to colonic inflammation (22).

Then how does TLR4 specifically participate in IBS disease progression through immune cells? In the intestine, TLR4 is expressed on antigen-presenting cells (e.g., macrophages and dendritic cells) and lymphocytes (65). In normal physiology, immune cell expression of TLR4 is required for B cell recruitment, dendritic cell maturation, and triggering of T cell responses to invading pathogens (18). A Mendelian randomization study shows a significant genetic correlation between immune cell phenotype and IBS (66). In IBS, alterations in lymphocyte populations, including B and T lymphocyte counts and activation levels, are associated with increased colonic MCs in IBS patients (67). Colonic mast cells are more numerous in IBS, and their activation degranulation can modulate visceral sensitivity and epithelial barrier function by releasing neuroactive mediators (53, 55, 68, 69). In clinical trials, mast cell stabilizers or histamine 1 receptor antagonists improved IBS symptoms and quality of life (20, 70). In an experiment of IBS supernatant-induced degranulation of BMMCs (bone marrow-derived MCs (BMMCs)), it was found that TLR4 activation led to degranulation and histamine production and that a TLR4 inhibitor (TAK-242) attenuated degranulation of BMMCs (71). In animal experiments, the potential mechanism of visceral hypersensitivity has also been found to involve the expression of TLR4 in MCs of colonic tissues (58). It indicates that TLR4 may influence intestinal function and visceral sensitization responses by regulating mast cell degranulation. However, no evidence of TLR4 regulation of other immune cells in IBS was found. Most of the studies were unclear in their observation of cell specificity and failed to provide a distinction between epithelial cells and immune cells expressing TLR4, thus preventing in-depth analysis of the specific mechanism (18).

### 4.2 TLR4 and intestinal barrier function

Increased intestinal permeability in patients with IBS ranges from 2% - 62% (72). The increased intestinal permeability in IBS is associated with abdominal pain and visceral sensitivity and exposes neurological and immune components to luminal microbes (73, 74). In IBS patients, TLR4 is strongly associated with barrier function-related genes, including protease-activated receptor 2, OCLN, and TJP1, suggesting potential functional relationships (75). Xi et al. found that overexpression of TLR4 led to down-regulation of TJP1 and OCLN, whereas inhibition of TLR4 expression led to up-regulation of TJP1 and OCLN in the IBS-D cell model (13). Singh P. et al. found that a high-FODMAP diet leads to colonic barrier loss and mast cell activation and that TLR4 receptors on MCs are critical for this high-FODMAP mediated loss of the colonic barrier (76). *In vivo* and *in vitro* experiments have revealed that activation of TLR4 increases intestinal permeability by down-regulating phosphorylated OCLN expression in the intestinal epithelial barrier, increasing myosin light chain kinase protein expression and kinase activity (77, 78). MUC2, a glycoprotein, forms the mucus layer of the intestinal barrier (79). *In vitro* experiment, silencing of TLR4 in the TLR4-expressing rat intestinal epithelioid cell line 6 induced MUC2 production, whereas overexpression of TLR4 in human Caco-2 cells, which generally do not express TLR4, resulted in the loss of their normal MUC2-producing phenotype (80). In the same *in vivo* experiments, increased permeability of the gut in villin-TLR4 mice (increased TLR4 signaling), about the significantly lower expression of epithelial cell-cell adhesion genes in colonic ECs, including junctional adhesion molecule A and cadherin-1, and a decreased depression of TJP1 although not significantly (81). In conclusion, aberrant expression of TLR4 is closely related to intestinal barrier function and is involved in barrier damage in IBS.

### 4.3 TLR4 and intestinal flora

In a healthy state, the gut microbiota interacts closely with intestinal epithelial cells and the immune system to regulate inflammation and maintain the development of intestinal barrier and immune system (82, 83). Intestinal dysbiosis, an imbalance in the intestinal microbiota due to various internal and external factors, is an important causative factor in IBS (72, 84). Several Meta-analyses of altered intestinal flora in patients with IBS have shown the presence of intestinal dysbiosis in patients with IBS, mainly characterized by lower levels of *lactobacilli* and *bifidobacteria* compared to healthy controls (85, 86). Flora dysbiosis may cause loss of intestinal integrity and increased intestinal permeability, which can lead to penetration of the epithelial barrier by bacterial products and metabolites, thereby triggering an inflammatory response (87). Increased intestinal permeability may also increase bacterial dissemination (81). In addition, intestinal dysbiosis affects intestinal motility, increases VHS, and regulates the gut-brain axis (88, 89). TLRs recognize specific microbial components of commensal and pathogenic bacteria and play a role in immune tolerance to commensal bacteria and defense against pathogens (90). Altered microbiota profiles may affect TLR expression and immune activation in IBS (30). Guo et al. found that the diversity of intestinal mucosal colonizing flora and the two dominant bacterial genera *(Mycobacterium avium* and *Clostridium* spp.) were significantly reduced in IBS-D patients compared with that of healthy people, while the mucosal immune-related receptors TLR2 and TLR4 were significantly over-expressed, and there was a correlation between the reduction of these two genera and the high expression of TLR (26). Similarly, another study found that TLR4 expression in IBS-D was negatively associated with the microbial relative abundance of the *Lactobacillus* and *Escherichia/Shigella* genera, whereas it was positively associated with the relative abundance of the genera *Megasphera* and *Sutterella* and the class *Betaproteobacteria* (27). Both studies suggest a correlation between TLR4 and gut flora. Bacterial invasion can activate pro-inflammatory responses through TLR4-induced TIRAP/MyD88 and TRAM/TRIF signaling cascades (91). Thus, intestinal dysbiosis may induce immune disorders by activating the natural and acquired immune systems by activating intestinal mucosal TLR4 proteins, triggering inflammation and ultimately leading to IBS-D (26). Targeting TLR4 may benefit by restoring epithelial function and changing the microbiota (81). Future studies are needed to explore the mechanisms of gut flora dysbiosis and the role of the TLR4 pathway in IBS disease.

### 4.4 TLR4 and visceral hypersensitivity

VHS, which refers to internal organs like the gastrointestinal tract exhibiting amplified perception of pain in response to stimuli, is a frequent complaint among individuals with IBS. The inflammatory consequences of TLR activation on glial cells (mainly microglia and astrocytes), sensory neurons, and other cell types affect injury perception processing and lead to pain (92). TLR4 expression in colonic tissues is associated with VHS reactions (58). Correlation analysis shows that TLR4 mRNA expression correlates with the intensity of abdominal pain in IBS-D patients (20). LPS Activation of TLR4 increases the production of pro-inflammatory cytokines, which activate visceral sensory neurons to induce visceral hypersensitivity (93). The results of eight studies have shown that VHS in IBS may be mediated through the TLR4, TLR4/NF-κB, TLR4/MyD88/NF-κB, TLR4/NF-κB/CBS, HMGB1/TLR4 pathways (12, 13, 35, 37–41). Genetically altered (i.e., TLR4 knockout and point-mutant) mice and rats with down-regulated TLR4 expression exhibit analgesia and low expression levels of cytokines, such as TNF-α and IL-1β (94). Tang et al. found that MS was associated with increased VHS, microglial TLR4, and inflammatory factors IL-1β and TNF-α expression in Tlr4 +/+ mice; however, MS did not alter VHS, IL-1β and TNF-α expression in Tlr4 -/- mice (12). Increased IL-1β and TNF-α proteins released by microglia via the TLR4/MyD88/NF-κB signaling pathway induced neonatal stress-induced VHS and pain (35). Administration of OT peripherally reduced VHS or visceral pain in human samples and animal models (39). OT pretreatment inhibited these increases as TLR4 signaling elicited a cellular response that released the downstream effectors MyD88, NF-κB, IL-1β, and TNF-α, thereby inducing VHS (39). TLR4 signaling and the pro-inflammatory cytokines TNF-α and IL-1β may be involved in neuroglial interactions in the pathogenesis of VHS reactions (41). TLR4 deficiency reduced visceral pain and prevented the development of chronic psychosocial stress-induced VHS. Administration of TLR4 antagonists, such as TAK-242 and CLI-095, counteracted chronic stress, neonatal colonic inflammation, and neonatal CRD-induced VHS (38, 40, 95). Furthermore, rats with a FODMAP diet resulted in impaired gut barrier function and increased sensitivity to colorectal distension, its LPS or fecal supernatants induced VHS, and this was blocked by small interfering RNA inhibition of TLR4 mRNA, suggesting that TLR4 activation by fecal LPS could mediate VHS (96). In conclusion, the generation of VHS is closely related to TLR4, NF-κB, and pro-inflammatory effects, which may be an essential way to improve abdominal pain in IBS patients.

### 4.5 TLR4 and defecation abnormalities

Abnormal defecation is one of the main symptoms of IBS, including changes in fecal water content and bowel motility. TLRs, especially TLR2 and TLR4, significantly affect post-infection and lipopolysaccharide-mediated regulation of gastrointestinal motility (97). The muscle contractility induced by acetylcholine was significantly lower in TLR2 (-/-) and TLR4 (-/-) concerning WT mice (98). Gastrointestinal motility was significantly delayed in mice that do not express TLR4 or Myd88 compared to wild-type mice (99). These studies suggest that TLR4 is involved in intestinal motility. In patients with IBS, a positive correlation was found between mRNA levels of TLR-4 and weekly stool frequency in IBS patients (30). High expression of TLR4 in the IBS model results in shorter bowel intervals, higher fecal water content, and greater urgency (13, 27).

### 4.6 TLR4 and psychosocial

IBS affects psychosocial factors, including general and gut-related anxiety, depression, and somatization (8, 100). Some of these affections are bidirectional, and psychosocial factors can aggravate IBS symptoms and the disease progression, and vice versa (101, 102). Up to the minute, two Mendelian randomization studies revealed a bidirectional causal relationship between IBS and cerebral cortex structures, confirming the two-way communication along the brain-gut axis (103, 104). Moreover, there are longitudinal interactions from childhood into adulthood. A Swedish prospective longitudinal birth-cohort study found that health-related quality of life deterioration and psychological distress of adolescence are associated with new cases of adult IBS, and undergoing an abdominal pain–related adolescent gut-brain interaction disorder is associated with new-onset adult psychological distress (105). Growing evidence suggests that TLRs are associated with the pathophysiology of major depressive disorder, among which multiple linear regression analysis revealed that TLR4 was an independent risk factor relating to the severity of major depression (106, 107). TLRs (including TLR4) are upregulated in major depressive disorder patients, while antidepressant treatment downregulates TLRs expression, suggesting that TLRs are critical mediators for antidepressant therapy (108). Clinical studies have found that patients with IBS and depression have higher levels of TLR4 expression and IL-6, accompanied by a decrease in IL-10, which indicates that TLR participates in the inflammation reaction in IBS and depression (31). Erick J et al. found that chronic restraint stress induces anxiety-like behaviors, while blockade of the HMGB1/TLR4 pathway reverses chronic restraint stress-induced anxiety-like behaviors (37). Some drugs can treat depression through TLR4 and its signaling pathway. XYS can improve depressive-like behavior in rats by suppressing the activation of the TLR4/NLRP3 inflammasome signaling pathway (57). In the LPS-induced depression model, the use of raspberry ketone supplementation can alleviate depressive behavior through mitigated gut inflammation by inhibiting the TLR-4/NF-κB pathway (109).

### 4.7 Corticotropin-releasing factor signaling systems and IBS

The development and worsening of symptoms in irritable bowel syndrome is known to be closely related to stress, which induces visceral hypersensitivity and altered colonic motility and plays a vital role in the pathophysiology of disease development (110, 111). CRF, expressed in the brain and colon, is a significant mediator of the brain-gut axis stress response and mediates stress-induced enhancement of colonic motility and VHS, suggesting that CRF is a critical component of IBS (112). CRF receptor subtype 1 (CRF1), CRF2, and TLR4 were found to be upregulated in peripheral blood samples of IBS patients, especially in patients with concomitant depression (31). Persistent activation of the CRF1 system at central or peripheral sites may be one of the underlying causes of diarrhea and abdominal pain symptoms in IBS (112). Colonic TLR4 expression is downregulated in CRF-deficient mice and is more susceptible to colitis (113). One study found that CRF induces VHS and colonic hyperpermeability via TLR4 and cytokine systems, and these changes are dependent on CRF1 (114). They also found that LPS-induced VHS is mediated through CRF, TLR4, and pro-inflammatory factor pathways (115). Moreover, LPS increases colonic CRF expression at the gene and protein level (116) and activates peripheral CRF receptors (115). The CRF-TLR4-inflammatory cytokine system also affects the gut microbiota. Evidence suggests that activation of the CRF-TLR4-inflammatory cytokine system is followed by impairment of the intestinal barrier, which may alter the microbiota (93). In conclusion, CRF and TLR4-pro-inflammatory cytokine signaling generates a vicious cycle of mutual activation, leading to intestinal barrier damage and ecological dysregulation, affecting intestinal motility, and inducing a visceral hypersensitivity response that leads to IBS symptoms.

### 4.8 MiRNAs involved in TLR4 regulation in IBS

MiRNAs regulate the pathophysiological mechanisms of IBS, and searching for relevant miRNA biomarkers as diagnostic and therapeutic candidates for IBS is a hot topic (117, 118). Only one publication reported that miR-16 in IBS regulates defecation intervals, stool water content, and VHS by targeting the TLR4/NF-κB/XIST pathway (13). It has been reported that miR-16 targets and inhibits TLR4 in the LPS-induced inflammatory pathway, and this alteration can be reversed by the lncRNA SNHG16 (119). In addition, miR-16 can down-regulate the expression of NF-κB, NLRP3, and other inflammatory factors by targeting TLR4, thereby attenuating inflammation in the LPS-induced acute lung injury model (120). Given that most miRNAs have many-to-many relationships with target genes, more studies are needed to clarify the molecular mechanisms of miRNAs in IBS on the TLR4 pathway.

### 4.9 TLR4 Single-nucleotide polymorphism and IBS

Genetics contributes to the development of IBS disease. As early as 2001, it was proposed that identical twins have a significantly higher concordance of IBS than dizygotic twins (121). SNPs are the most common type of sequence variation in genomes. It has been shown that TLR9 rs5743836 (A/g) gene polymorphism may be associated with IBS-D phenotype (122). However, this review did not retrieve complete text reports on TLR4 gene polymorphism in IBS. A study of SNPs in the TLR4 gene in inflammatory bowel disease shows that TLR4 D299G polymorphism is significantly associated with inflammatory bowel disease in North Indian populations and regulates the transcription of inflammatory cytokines during ulcerative colitis, leading to abnormal immune responses (123). The results suggest that SNPs in TLR4 are associated with immune inflammation, which is the primary pathogenesis of IBS. Its role in IBS needs to be clarified, and future studies can look for its role in IBS regarding TLR4 gene polymorphisms.

### 4.10 TLR4’s feasibility as a potential therapeutic target

We included 18 studies in this review, which reported specific treatments for IBS by targeting TLR4. All these studies found that inhibition of TLR4 was one of the essential mechanisms underlying the improved IBS exerted by specific treatments. Multiple studies have found that chronic psychosocial stress or early-life stress impacts susceptibility to IBS by modulating TLR4, etc. (19, 37, 124, 125). KD might be beneficial in psychiatric disorders like stress, anxiety, depression, mood disorders, etc., given its ability to remodel the gut microbiota and antioxidant and anti-inflammatory effects, consequently impacting the brain-gut axis (126, 127). Chimienti et al. found that feeding animals with KD can reduce inflammation and oxidative stress, restore mitochondrial function and baseline autophagy, and thus reduce the harmful effects of stress in an animal model of IBS (43). In addition to diet therapy, EA and moxibustion therapy also have significant therapeutic effects on IBS (128, 129). These therapies may be practical to decrease patients’ pain, and fewer side effects will be sought.

Regarding the mechanism, the study showed that moxibustion improved diarrhea symptoms and VHS and alleviated inflammation of IBS-D through inhibited TLR4/MyD88/NF-κB signaling pathway (44). At the same time, EA-reduced visceral sensitivity of IBS may be involved in the suppression of TLR4 expression in the MCs of colonic tissues, which inhibits mast cell activation in colonic tissues and reduces the levels of inflammatory factors in serum that participate in the process of VHS (58). Multiple systematic reviews and meta-analyses have shown that herbal medicine effectively relieves IBS symptoms (130–132). TLR4 was the most critical type of TLRs regulated by phytochemicals (133). Herbal compound prescriptions, such as Wumei Pill, STW 5-II, QingHuaZhiXie Prescription, Sancao Lichang Decoction, CKF, Xiaoyaosan, improvement of inflammation, defecation abnormalities, VHS and depressive behavior in IBS, via suppressed TLR4, TLR4/MyD88/NF-κB or TLR4/NF-κB/NLRP3 signal pathway (45–47, 54, 55, 57). Flavonoids, mostly found as natural pigments in fruits, vegetables, and seeds of edible plants, have significant therapeutic activities, such as anti-inflammatory and antioxidant effects, and enhance intestinal barrier function (133–135). GSE, LE, and Apigenin, natural flavonoids, could diminish inflammation, maintain tight junction integrity, and improve visceral sensitization and colonic hypermobility with IBS by inhibiting TLR4 and TLR4/MyD88/NF-κB pathway (42, 51, 53). Other herbs that act on TLR4, such as SHE and BHS, may also improve IBS symptoms (48, 56). Increasingly, evidence has shown that gut microbiota dysbiosis plays a vital role in IBS pathogenesis (86, 136). Probiotic use may offer particular utility in managing IBS through its metabolic activity, immunomodulatory, and cross-feeding effects (137). LC-DG and its PB, Sb, and probiotic combination LT could decrease TLR4, attenuate inflammation, improve colonic hypermotility, and prevent epithelial barrier impairment of IBS (24, 50, 52). In addition to probiotics, MT is also closely linked to the gut microbiota, mitigated colonic microbiota dysbiosis, and intestinal inflammation in IBS animal models by inhibiting the activation of the TLR4/NF-κB pathway (49). Overall, the 18 studies suggest that multiple therapies targeting TLR4 can reduce IBS-related symptoms. TLR4 is viable as a potential therapeutic target.

### 4.11 The relationship between TLR4 and IBS

TLR4 may play a vital role in the pathogenesis of IBS, and its abnormal activation may lead to dysregulation of the intestinal inflammatory response, which in turn affects the intestinal function, leading to hypersensitivity of the intestinal nervous system and aggravation of abdominal pain and discomfort. The structure of the intestinal flora of patients with BS may change, and the overgrowth of certain bacteria may lead to the activation of TLR4, which in turn may further affect the balance of the intestinal flora, forming a vicious circle and exacerbating the symptoms of IBS. Polymorphisms in the TLR4 gene may be associated with the risk of developing IBS. In addition, environmental factors such as diet and stress may also indirectly affect the development and symptoms of IBS by influencing the expression and function of TLR4. From the current evidence, changes in TLR4 and the development of IBS may be causative, and more clinical and basic studies are needed to elucidate the exact causal relationship between TLR4 and IBS.

## 5 Conclusion

TLR4 is significantly up-regulated in IBS, correlating with clinical manifestations, and is accompanied by up-regulation of pro-inflammatory factors and down-regulation of anti-inflammatory factors. Pathogenesis involved in IBS, such as intestinal barrier damage, intestinal dysbiosis, abnormal intestinal peristalsis, increased visceral sensitization, and anxiety behaviors, are related to the interactions among TLR4, the NF-κB pathway, the pro-inflammatory effects, and the CRFs. Various therapies such as herbs, acupuncture, probiotics, and their associated PB can treat IBS by targeting TLR4 and its pathway. In conclusion, TLR4 may be a promising target for treating IBS, and more clinical studies will be needed to evaluate therapeutic approaches targeting this pathway.

## Data Availability

The original contributions presented in the study are included in the article/supplementary material. Further inquiries can be directed to the corresponding author/s.

## References

[1] Ford AC Sperber AD Corsetti M Camilleri M . Irritable bowel syndrome. Lancet. (2020) 396:1675–88. doi: 10.1016/S0140-6736(20)31548-8 33049223

[2] Drossman DA . Functional gastrointestinal disorders: history, pathophysiology, clinical features, and rome IV. Gastroenterology. 150:1262–79. doi: 10.1053/j.gastro.2016.02.032 27144617

[3] Sperber AD Bangdiwala SI Drossman DA Ghoshal UC Simren M Tack J . Worldwide prevalence and burden of functional gastrointestinal disorders, results of rome foundation global study. Gastroenterology. (2021) 160:99–114.e3. doi: 10.1038/ajg.2010.40 32294476

[4] Chang JY Locke RGI McNally MA Halder SL Schleck CD Zinsmeister AR . Impact of functional gastrointestinal disorders on survival in the community. Off J Am Coll Gastroenterol ACG. (2010) 105:822–32. doi: 10.1186/s12955-017-0611-2 PMC2887253 20160713

[5] Buono JL Carson RT Flores NM . Health-related quality of life, work productivity, and indirect costs among patients with irritable bowel syndrome with diarrhea. Health Qual Life Outcomes. (2017) 15:35. 28196491 10.1186/s12955-017-0611-2 PMC5310011

[6] Frändemark Å Törnblom H Jakobsson S Simrén M . Work productivity and activity impairment in irritable bowel syndrome (IBS): A multifaceted problem. Off J Am Coll Gastroenterol ACG. (2018) 113:1540–9. doi: 10.1038/s41395-018-0262-x 30254230

[7] Sugaya N . Work-related problems and the psychosocial characteristics of individuals with irritable bowel syndrome: an updated literature review. Biopsychosoc Med. (2024) 18:12. doi: 10.1186/s13030-024-00309-5 38750514 PMC11094939

[8] Patel P Bercik P Morgan DG Bolino C Pintos-Sanchez MI Moayyedi P . Irritable bowel syndrome is significantly associated with somatisation in 840 patients, which may drive bloating. Aliment Pharmacol Ther. (2015) 41:449–58. doi: 10.1111/apt.2015.41.issue-5 25586008

[9] Goodoory VC Ng CE Black CJ Ford AC . Direct healthcare costs of Rome IV or Rome III-defined irritable bowel syndrome in the United Kingdom. Aliment Pharmacol Ther. (2022) 56:110–20. doi: 10.1111/apt.16939 PMC9325446 35491477

[10] Canavan C West J Card T . Review article: the economic impact of the irritable bowel syndrome. Aliment Pharmacol Ther. (2014) 40:1023–34. doi: 10.1111/apt.2014.40.issue-9 25199904

[11] Bosman MHMA Weerts ZZRM Snijkers JTW Vork L Mujagic Z Masclee AAM . The socioeconomic impact of irritable bowel syndrome: an analysis of direct and indirect health care costs. Clin Gastroenterol Hepatol. (2023) 21:2660–9. doi: 10.1016/j.cgh.2023.01.017 36731587

[12] Tang HL Zhang G Ji NN Du L Chen BB Hua R . Toll-like receptor 4 in paraventricular nucleus mediates visceral hypersensitivity induced by maternal separation. Front Pharmacol. (2017) 8:309/full. doi: 10.3389/fphar.2017.00309/full 28611665 PMC5447361

[13] Xi M Zhao P Li F Bao H Ding S Ji L . MicroRNA-16 inhibits the TLR4/NF-κB pathway and maintains tight junction integrity in irritable bowel syndrome with diarrhea. J Biol Chem. (2022) 298:102461. doi: 10.1016/j.jbc.2022.102461 36067883 PMC9647533

[14] Leulier F Lemaitre B . Toll-like receptors — taking an evolutionary approach. Nat Rev Genet. (2008) 9:165–78. doi: 10.1038/nrg2303 18227810

[15] Giambra V Pagliari D Rio P Totti B Di Nunzio C Bosi A . Gut microbiota, inflammatory bowel disease, and cancer: the role of guardians of innate immunity. Cells. (2023) 12:2654. doi: 10.3390/cells12222654 37998389 PMC10669933

[16] Abreu MT . Toll-like receptor signalling in the intestinal epithelium: how bacterial recognition shapes intestinal function. Nat Rev Immunol. (2010) 10:131–44. doi: 10.1038/nri2707 20098461

[17] Bogunovic M Davé SH Tilstra JS Chang DTW Harpaz N Xiong H . Enteroendocrine cells express functional Toll-like receptors. Am J Physiol Gastrointest Liver Physiol. (2007) 292:G1770–1783. doi: 10.1152/ajpgi.00249.2006 PMC3203538 17395901

[18] Bruning EE Coller JK Wardill HR Bowen JM . Site-specific contribution of Toll-like receptor 4 to intestinal homeostasis and inflammatory disease. J Cell Physiol. (2021) 236:877–88. doi: 10.1002/jcp.v236.2 32730645

[19] Zhou GQ Huang MJ Yu X Zhang NN Tao S Zhang M . Early life adverse exposures in irritable bowel syndrome: new insights and opportunities. Front Pediatr. (2023) 11:1241801/full. doi: 10.3389/fped.2023.1241801/full 37732013 PMC10507713

[20] Lobo B Ramos L Martínez C Guilarte M González-Castro AM Alonso-Cotoner C . Downregulation of mucosal mast cell activation and immune response in diarrhoea-irritable bowel syndrome by oral disodium cromoglycate: A pilot study. United Eur Gastroenterol J. (2017) 5:887–97. doi: 10.1177/2050640617691690 PMC5625876 29026603

[21] Hyo-Jin K Kim H Jeong-Hyung L Hwangbo C . Toll-like receptor 4 (TLR4): new insight immune and aging. Immun Ageing. (2023) 20:1–11. doi: 10.1186/s12979-023-00383-3 38001481 PMC10668412

[22] Belmonte L Youmba SB Bertiaux-Vandaële N Antonietti M Lecleire S Zalar A . Role of toll like receptors in irritable bowel syndrome: differential mucosal immune activation according to the disease subtype. PloS One. (2012) 7:e42777. doi: 10.1371/journal.pone.0042777 23028414 PMC3461726

[23] Brint EK MacSharry J Fanning A Shanahan F Quigley EMM . Differential expression of toll-like receptors in patients with irritable bowel syndrome. Off J Am Coll Gastroenterol ACG. (2011) 106:329–36. doi: 10.1038/ajg.2010.438 21102570

[24] Compare D Rocco A Coccoli P Angrisani D Sgamato C Iovine B . Lactobacillus casei DG and its postbiotic reduce the inflammatory mucosal response: an ex-vivo organ culture model of post-infectious irritable bowel syndrome. BMC Gastroenterol. (2017) 17:53. doi: 10.1186/s12876-017-0605-x 28410580 PMC5391611

[25] Dlugosz A Zakikhany K Acevedo N D’Amato M Lindberg G . Increased expression of toll-like receptors 4, 5, and 9 in small bowel mucosa from patients with irritable bowel syndrome. BioMed Res Int. (2017) 2017:9624702. doi: 10.1155/2017/9624702 28246611 PMC5303582

[26] Guo W Liu P Dong L Wang J . The correlation study between the changes of intestinal mucosa predominant bacteria and Toll-like receptor 2, Toll-like receptor 4 gene expressions in diarrhea-predominant irritable bowel syndrome pafients. Chin J Intern Med. (2016) 55:541–3. doi: 10.3760/cma.j.issn.0578-1426.2016.07.011 27373290

[27] Jalanka J Lam C Bennett A Hartikainen A Crispie F Finnegan LA . Colonic Gene Expression and Fecal Microbiota in Diarrhea-predominant Irritable Bowel Syndrome: Increased Toll-like Receptor 4 but Minimal Inflammation and no Response to Mesalazine. J Neurogastroenterol Motil. (2021) 27:279–91. doi: 10.5056/jnm20205 PMC8026366 33795545

[28] Koçak E Akbal E Köklü S Ergül B Can M . The colonic tissue levels of TLR2, TLR4 and nitric oxide in patients with irritable bowel syndrome. Intern Med Tokyo Jpn. (2016) 55:1043–8. doi: 10.2169/internalmedicine.55.5716 27150852

[29] Linsalata M Riezzo G D’Attoma B Clemente C Orlando A Russo F . Noninvasive biomarkers of gut barrier function identify two subtypes of patients suffering from diarrhoea predominant-IBS: a case-control study. BMC Gastroenterol. (2018) 18:167. doi: 10.1186/s12876-018-0888-6 30400824 PMC6219148

[30] Shukla R Ghoshal U Ranjan P Ghoshal UC . Expression of toll-like receptors, pro-, and anti-inflammatory cytokines in relation to gut microbiota in irritable bowel syndrome: the evidence for its micro-organic basis. J Neurogastroenterol Motil. (2018) 24:628–42. doi: 10.5056/jnm18130 PMC6175562 30347939

[31] Jizhong S Qiaomin W Chao W Yanqing L . Corticotropin-releasing factor and toll-like receptor gene expression is associated with low-grade inflammation in irritable bowel syndrome patients with depression. Gastroenterol Res Pract. (2016) 2016:7394924. doi: 10.1155/2016/7394924 27478433 PMC4960335

[32] Wang XM Liu YL . Signal transduction pathway of colonic Toll-like receptor 4 in patients with irritable bowel syndrome. Zhonghua Yi Xue Za Zhi. (2008) 88:3415–7. doi: 10.3321/j.issn:0376-2491.2008.48.008 19159572

[33] Yoshimoto T Oshima T Huang X Tomita T Fukui H Miwa H . Microinflammation in the intestinal mucosa and symptoms of irritable bowel syndrome. J Gastroenterol. (2022) 57:62–9. doi: 10.1007/s00535-021-01838-4 34854984

[34] Bao LL Cui LH . Exprossion of TLR4 and NF-κB in rats model induced with IBS-D and its mechanism. Med J Chin Peoples Lib Army. (2019) 44:648–51. doi: 10.11855/j.issn.0577-7402.2019.08.04

[35] Chen ZY Zhang XW Yu L Hua R Zhao XP Qin X . Spinal toll-like receptor 4-mediated signalling pathway contributes to visceral hypersensitivity induced by neonatal colonic irritation in rats. Eur J Pain. (2015) 19:176–86. doi: 10.1002/ejp.534 24842692

[36] He X Cui LH Wang XH Yan ZH Li C Gong SD . Modulation of inflammation by toll-like receptor 4/nuclear factor-kappa B in diarrhea-predominant irritable bowel syndrome. Oncotarget. (2017) 8:113957–65. doi: 10.18632/oncotarget.23045 PMC5768377 29371960

[37] Rodriguez-Palma EJ Velazquez-Lagunas I Salinas-Abarca AB Vidal-Cantu GC Escoto-Rosales MJ Castaneda-Corral G . Spinal alarmin HMGB1 and the activation of TLR4 lead to chronic stress-induced nociceptive hypersensitivity in rodents. Eur J Pharmacol. (2023) 952:175804. doi: 10.1016/j.ejphar.2023.175804 37244377

[38] Wu J Li T Mao G Cha X Fei S Miao B . The involvement of Pellino-1 downregulation in the modulation of visceral hypersensitivity via the TLR4/NF-κB pathway in the rat fastigial nucleus. Neurosci Lett. (2022), 787:136815. doi: 10.1016/j.neulet.2022.136815 35901910

[39] Xu S Qin B Shi A Zhao J Guo X Dong L . Oxytocin inhibited stress induced visceral hypersensitivity, enteric glial cells activation, and release of proinflammatory cytokines in maternal separated rats. Eur J Pharmacol. (2018) 818:578–84. doi: 10.1016/j.ejphar.2017.11.018 29162434

[40] Yuan B Tang WH Lu LJ Zhou Y Zhu HY Zhou YL . TLR4 upregulates CBS expression through NF-&kappa;B activation in a rat model of irritable bowel syndrome with chronic visceral hypersensitivity. World J Gastroenterol. (2015) 21:8615–28. doi: 10.3748/wjg.v21.i28.8615 PMC4515842 26229403

[41] Zhang G Yu L Chen ZY Zhu JS Hua R Qin X . Activation of corticotropin-releasing factor neurons and microglia in paraventricular nucleus precipitates visceral hypersensitivity induced by colorectal distension in rats. Brain Behav Immun. (2016) 55:93–104. doi: 10.1016/j.bbi.2015.12.022 26743854

[42] Arie H Nozu T Miyagishi S Ida M Izumo T Shibata H . Grape seed extract eliminates visceral allodynia and colonic hyperpermeability induced by repeated water avoidance stress in rats. Nutrients. (2019) 11:2646. doi: 10.3390/nu11112646 31689935 PMC6893525

[43] Chimienti G Orlando A Lezza AMS D’Attoma B Notarnicola M Gigante I . The ketogenic diet reduces the harmful effects of stress on gut mitochondrial biogenesis in a rat model of irritable bowel syndrome. Int J Mol Sci. (2021) 22:3498. doi: 10.3390/ijms22073498 33800646 PMC8037144

[44] Chu HR Wang Y Tong L Wu SB Wu LB Li N . Effect of moxibustion on TLR4/MyD88/NF-κB signaling pathway in colon of diarrhea-predo-minant irritable bowel syndrome rats. Zhen Ci Yan Jiu Acupunct Res. (2020) 45:633–9. doi: 10.13702/j.1000-0607.190950 32869573

[45] Ding X Sun X Wang Z Zheng Q Yu X Hao W . The effects of Wumei pill on TLRs/NF-kB signaling pathway in rats with diarrhea-predominant irritable bowel syndrome. Pak J Zool. (2019) 51:57–65. doi: 10.17582/journal.pjz/2019.51.1.57.65

[46] Elbadawi M Ammar RM Rabini S Klauck SM Efferth T . Modulation of intestinal corticotropin-releasing hormone signaling by the herbal preparation STW 5-II: possible mechanisms for irritable bowel syndrome management. Pharmaceuticals. (2022) 15:1121. doi: 10.3390/ph15091121 36145342 PMC9504045

[47] Huang H Zhao P Xi M Li F Ji L . Mechanism of qingHuaZhiXie prescription regulating TLR4-IECs pathway in the intervention of diarrhea predominant irritable bowel syndrome. Evid Based Complement Alternat Med. (2021) 2021:5792130. doi: 10.1155/2021/5792130 34795785 PMC8594983

[48] Ruiz-Malagón AJ Rodríguez-Sanchez MJ Rodríguez-Sojo MJ Vezza T Pischel I Algieri F . Intestinal anti-inflammatory and visceral analgesic effects of a Serpylli herba extract in an experimental model of irritable bowel syndrome in rats. Front Pharmacol. (2022) 13:967644/full. doi: 10.3389/fphar.2022.967644/full 36120292 PMC9479127

[49] Lin R Wang Z Cao J Gao T Dong Y Chen Y . Role of melatonin in murine “restraint stress”-induced dysfunction of colonic microbiota. J Microbiol. (2021) 59:500–12. doi: 10.1007/s12275-021-0305-7 33630247

[50] Liu J Ren H Yuan F Shao M Luo H . The effects of Saccharomyces boulardii on rat colonic hypermotility induced by repeated water avoidance stress and the potential mechanism. PeerJ. (2022) 10:e14390. doi: 10.7717/peerj.14390 36438584 PMC9695494

[51] Maqoud F Orlando A Tricarico D Antonacci M Di Turi A Giannelli G . Anti-inflammatory effects of a novel acetonitrile–water extract of lens culinaris against LPS-induced damage in caco-2 cells. Int J Mol Sci. (2024) 25:3802. doi: 10.3390/ijms25073802 38612611 PMC11011527

[52] Nébot-Vivinus M Harkat C . Multispecies probiotic protects gut barrier function in experimental models. World J Gastroenterol. (2014) 20:6832–43. doi: 10.3748/wjg.v20.i22.6832 PMC4051923 24944474

[53] Xia Y Peng S Lin M Duan H Yuan F Shao M . Apigenin attenuates visceral hypersensitivity in water avoidance stress rats by modulating the microbiota-gut-brain axis and inhibiting mast cell activation. BioMed Pharmacother. (2023) 167:115562. doi: 10.1016/j.biopha.2023.115562 37801900

[54] Zhang P Ma Y Wang Z Tang D . The effect and mechanism of sancao lichang decoction on diarrhea- predominant irritable bowel syndrome by regulating tlr4/myd88/nf-Kb pathway. doi: 10.2174/1386207326666230301104248 36856180

[55] Zhang S Tian D Xia Z Yang F Chen Y Yao Z . Chang-Kang-Fang alleviates diarrhea predominant irritable bowel syndrome (IBS-D) through inhibiting TLR4/NF-κB/NLRP3 pathway. J Ethnopharmacol. (2024) 330:118236. doi: 10.1016/j.jep.2024.118236 38670405

[56] Zhu HM Li L Li SY Yan Q Li F . Effect of water extract from Berberis heteropoda Schrenk roots on diarrhea-predominant irritable bowel syndrome by adjusting intestinal flora. J Ethnopharmacol. (2019) 237:182–91. doi: 10.1016/j.jep.2019.03.045 30902748

[57] Zhu HZ Liang YD Hao WZ Ma QY Li XJ Li YM . Xiaoyaosan exerts therapeutic effects on the colon of chronic restraint stress model rats via the regulation of immunoinflammatory activation induced by the TLR4/NLRP3 inflammasome signaling pathway. Evid-Based Complement Altern Med ECAM. (2021) 2021:6673538. doi: 10.1155/2021/6673538 PMC7810549 33505499

[58] Yang J Shang B Shi H Zhu S Lu G Dai F . The role of toll-like receptor 4 and mast cell in the ameliorating effect of electroacupuncture on visceral hypersensitivity in rats. Neurogastroenterol Motil. (2019) 31:e13583. doi: 10.1111/nmo.2019.31.issue-6 30916854

[59] Seyedmirzaee S Hayatbakhsh MM Ahmadi B Baniasadi N Rafsanjani AMB Nikpoor AR . Serum immune biomarkers in irritable bowel syndrome. Clin Res Hepatol Gastroenterol. (2016) 40:631–7. doi: 10.1016/j.clinre.2015.12.013 26850360

[60] Choghakhori R Abbasnezhad A Hasanvand A Amani R . Inflammatory cytokines and oxidative stress biomarkers in irritable bowel syndrome: Association with digestive symptoms and quality of life. CYTOKINE. (2017) 93:34–43. doi: 10.1016/j.cyto.2017.05.005 28506572

[61] Bashashati M Moradi M Sarosiek I . Interleukin-6 in irritable bowel syndrome: A systematic review and meta analysis of IL-6 (-G174C) and circulating IL-6 levels. CYTOKINE. (2017) 99:132–8. doi: 10.1016/j.cyto.2017.08.017 28886490

[62] Liebregts T Adam B Bredack C Roth A Heinzel S Lester S . Immune activation in patients with irritable bowel syndrome. GASTROENTEROLOGY. (2007) 132:913–20. doi: 10.1053/j.gastro.2007.01.046 17383420

[63] Medzhitov R Preston-Hurlburt P Janeway CA . A human homologue of the Drosophila Toll protein signals activation of adaptive immunity. Nature. (1997) 388:394–7. doi: 10.1038/41131 9237759

[64] Liu T Zhang L Joo D Sun SC . NF-κB signaling in inflammation. Signal Transduct Target Ther. (2017) 2:1–9. doi: 10.1038/sigtrans.2017.23 PMC5661633 29158945

[65] Jilling T Simon D Lu J Meng FJ Li D Schy R . The roles of bacteria and TLR4 in rat and murine models of necrotizing enterocolitis. J Immunol Baltim Md. (1950) 177:3273–82. doi: 10.4049/jimmunol.177.5.3273 PMC2697969 16920968

[66] Chai WH Ma Y Li JJ Guo F Wu YZ Liu JW . Immune cell signatures and causal association with irritable bowel syndrome: A mendelian randomization study. World J Clin Cases. (2024) 12:3094–104. doi: 10.12998/wjcc.v12.i17.3094 PMC11185378 38898868

[67] Hasler WL Grabauskas G Singh P Owyang C . Mast cell mediation of visceral sensation and permeability in irritable bowel syndrome. Neurogastroenterol Motil. (2022) 34:e14339. doi: 10.1111/nmo.14339 35315179 PMC9286860

[68] Barbara G Stanghellini V De Giorgio R Cremon C Cottrell GS Santini D . Activated mast cells in proximity to colonic nerves correlate with abdominal pain in irritable bowel syndrome. Gastroenterology. (2004) 126:693–702. doi: 10.1053/j.gastro.2003.11.055 14988823

[69] Bashashati M Moossavi S Cremon C Barbaro MR Moraveji S Talmon G . Colonic immune cells in irritable bowel syndrome: A systematic review and meta-analysis. Neurogastroenterol Motil. (2018) 30:e13192. doi: 10.1111/nmo.2018.30.issue-1 28851005

[70] Klooker TK Braak B Koopman KE Welting O Wouters MM van der Heide S . The mast cell stabiliser ketotifen decreases visceral hypersensitivity and improves intestinal symptoms in patients with irritable bowel syndrome. Gut. (2010) 59:1213–21. doi: 10.1136/gut.2010.213108 20650926

[71] Shimbori C De Palma G Baerg L Lu J Verdu EF Reed DE . Gut bacteria interact directly with colonic mast cells in a humanized mouse model of IBS. Gut Microbes. (2022) 14:2105095. doi: 10.1080/19490976.2022.2105095 35905313 PMC9341375

[72] JohnBritto JS Di Ciaula A Noto A Cassano V Sciacqua A Khalil M . Gender-specific insights into the irritable bowel syndrome pathophysiology. Focus on gut dysbiosis and permeability. Eur J Intern Med. (2024) 125:10–8. doi: 10.1016/j.ejim.2024.03.011 38467533

[73] King AJ Chang L Li Q Liu L Zhu Y Pasricha PJ . NHE3 inhibitor tenapanor maintains intestinal barrier function, decreases visceral hypersensitivity, and attenuates TRPV1 signaling in colonic sensory neurons. Am J Physiol-Gastrointest LIVER Physiol. (2024) 326:G543–54. doi: 10.1152/ajpgi.00233.2023 PMC11376972 38252683

[74] Long Y Du L Kim JJ Chen B Zhu Y Zhang Y . MLCK-mediated intestinal permeability promotes immune activation and visceral hypersensitivity in PI-IBS mice. Neurogastroenterol Motil. (2018) 30:e13348. doi: 10.1111/nmo.2018.30.issue-9 29644768

[75] Polster A Öhman L Tap J Derrien M Le Nevé B Sundin J . A novel stepwise integrative analysis pipeline reveals distinct microbiota-host interactions and link to symptoms in irritable bowel syndrome. Sci Rep. (2021) 11:5521. doi: 10.1038/s41598-021-84686-9 33750831 PMC7943560

[76] Singh P Grabauskas G Zhou SY Gao J Zhang Y Owyang C . High FODMAP diet causes barrier loss via lipopolysaccharide-mediated mast cell activation. JCI Insight. (2021) 6. doi: 10.1172/jci.insight.146529 PMC8663790 34618688

[77] Li X Wang C Nie J Lv D Wang T Xu Y . Toll-like receptor 4 increases intestinal permeability through up-regulation of membrane PKC activity in alcoholic steatohepatitis. ALCOHOL. (2013) 47:459–65. doi: 10.1016/j.alcohol.2013.05.004 23871536

[78] Nighot M Al-Sadi R Guo S Rawat M Nighot P Watterson MD . Lipopolysaccharide-induced increase in intestinal epithelial tight permeability is mediated by toll-like receptor 4/myeloid differentiation primary response 88 (MyD88) activation of myosin light chain kinase expression. Am J Pathol. (2017) 187:2698–710. doi: 10.1016/j.ajpath.2017.08.005 PMC5718096 29157665

[79] Schoultz I Keita AV . The intestinal barrier and current techniques for the assessment of gut permeability. CELLS. (2020) 9:1909. doi: 10.3390/cells9081909 32824536 PMC7463717

[80] Sodhi CP Neal MD Siggers R Sho S Ma C Branca MF . Intestinal epithelial toll-like receptor 4 regulates goblet cell development and is required for necrotizing enterocolitis in mice. Gastroenterology. (2012) 143:708–U234. doi: 10.1053/j.gastro.2012.05.053 22796522 PMC3584415

[81] Dheer R Santaolalla R Davies JM Lang JK Phillips MC Pastorini C . Intestinal epithelial toll-like receptor 4 signaling affects epithelial function and colonic microbiota and promotes a risk for transmissible colitis. Infect Immun. (2016) 84:798–810. doi: 10.1128/IAI.01374-15 26755160 PMC4771346

[82] Van de Wiele T Van Praet JT Marzorati M Drennan MB Elewaut D . How the microbiota shapes rheumatic diseases. Nat Rev Rheumatol. (2016) 12:398–411. doi: 10.1038/nrrheum.2016.85 27305853

[83] Takiishi T Fenero CIM Câmara NOS . Intestinal barrier and gut microbiota: Shaping our immune responses throughout life. Tissue Barriers. (2017) 5:e1373208. doi: 10.1080/21688370.2017.1373208 28956703 PMC5788425

[84] Shaikh SD Sun N Canakis A Park WY Weber HC . Irritable bowel syndrome and the gut microbiome: A comprehensive review. J Clin Med. (2023) 12:2558. doi: 10.3390/jcm12072558 37048642 PMC10095554

[85] Liu HN Wu H Chen YZ Chen YJ Shen XZ Liu TT . Altered molecular signature of intestinal microbiota in irritable bowel syndrome patients compared with healthy controls: A systematic review and meta-analysis. Dig LIVER Dis. (2017) 49:331–7. doi: 10.1016/j.dld.2017.01.142 28179092

[86] Wang L Alammar N Singh R Nanavati J Song Y Chaudhary R . Gut microbial dysbiosis in the irritable bowel syndrome: A systematic review and meta-analysis of case-control studies. J Acad Nutr Diet. (2020) 120:565–86. doi: 10.1016/j.jand.2019.05.015 31473156

[87] Linsalata M Riezzo G Clemente C D’Attoma B Russo F . Noninvasive biomarkers of gut barrier function in patients suffering from diarrhea predominant-IBS: an update. Dis Markers. (2020) 2020:2886268. doi: 10.1155/2020/2886268 33110455 PMC7582069

[88] Principi N Cozzali R Farinelli E Brusaferro A Esposito S . Gut dysbiosis and irritable bowel syndrome: The potential role of probiotics. J Infect. (2018) 76:111–20. doi: 10.1016/j.jinf.2017.12.013 29291933

[89] Wollny T Daniluk T Piktel E Wnorowska U Bukłaha A Głuszek K . Targeting the gut microbiota to relieve the symptoms of irritable bowel syndrome. Pathogens. (2021) 10:1545. doi: 10.3390/pathogens10121545 34959500 PMC8705654

[90] de Kivit S Tobin MC Forsyth CB Keshavarzian A Landay AL . Regulation of intestinal immune responses through TLR activation: implications for pro- and prebiotics. Front Immunol. (2014) 5:60. doi: 10.3389/fimmu.2014.00060 24600450 PMC3927311

[91] Dmytriv TR Storey KB Lushchak VI . Intestinal barrier permeability: the influence of gut microbiota, nutrition, and exercise. Front Physiol. (2024) 15:1380713/full. doi: 10.3389/fphys.2024.1380713/full 39040079 PMC11260943

[92] Liu X Yang W Zhu C Sun S Wu S Wang L . Toll-like receptors and their role in neuropathic pain and migraine. Mol BRAIN. (2022) 15:73. doi: 10.1186/s13041-022-00960-5 35987639 PMC9392297

[93] Nozu T Okumura T . Pathophysiological commonality between irritable bowel syndrome and metabolic syndrome: role of corticotropin-releasing factor-toll-like receptor 4-proinflammatory cytokine signaling. J Neurogastroenterol Motil. (2022) 28:173–84. doi: 10.5056/jnm21002 PMC8978123 35189599

[94] Tanga FY Nutile-McMenemy N DeLeo JA . The CNS role of Toll-like receptor 4 in innate neuroimmunity and painful neuropathy. Proc Natl Acad Sci U S A. (2005) 102:5856–61. doi: 10.1073/pnas.0501634102 PMC556308 15809417

[95] Tramullas M Finger BC Moloney RD Golubeva AV Moloney G Dinan TG . Toll-like receptor 4 regulates chronic stress-induced visceral pain in mice. Biol Psychiatry. (2014) 76:340–8. doi: 10.1016/j.biopsych.2013.11.004 24331544

[96] Zhou SY Gillilland M Wu X Leelasinjaroen P Zhang G Zhou H . FODMAP diet modulates visceral nociception by lipopolysaccharide-mediated intestinal inflammation and barrier dysfunction. J Clin Invest. (2018) 128:267–80. doi: 10.1172/JCI92390 PMC5749529 29202473

[97] Nam Y Min YS Sohn UD . Recent advances in pharmacological research on the management of irritable bowel syndrome. Arch Pharm Res. (2018) 41:955–66. doi: 10.1007/s12272-018-1068-5 30132170

[98] Layunta E Forcén R Grasa L . TLR2 and TLR4 modulate mouse ileal motility by the interaction with muscarinic and nicotinic receptors. Cells. (2022) 11:1791. doi: 10.3390/cells11111791 35681486 PMC9180263

[99] Anitha M Vijay-Kumar M Sitaraman SV Gewirtz AT Srinivasan S . Gut microbial products regulate murine gastrointestinal motility via toll-like receptor 4 signaling. Gastroenterology. (2012) 143:1006. doi: 10.1053/j.gastro.2012.06.034 22732731 PMC3458182

[100] Black CJ Yiannakou Y Houghton LA Ford AC . Epidemiological, clinical, and psychological characteristics of individuals with self-reported irritable bowel syndrome based on the rome IV vs rome III criteria. Clin Gastroenterol Hepatol. (2020) 18:392–398.e2. doi: 10.1016/j.cgh.2019.05.037 31154027

[101] Koloski NA Jones M Kalantar J Weltman M Zaguirre J Talley NJ . The brain-gut pathway in functional gastrointestinal disorders is bidirectional: a 12-year prospective population-based study. GUT. (2012) 61:1284–90. doi: 10.1136/gutjnl-2011-300474 22234979

[102] Koloski NA Jones M Talley NJ . Evidence that independent gut-to-brain and brain-to-gut pathways operate in the irritable bowel syndrome and functional dyspepsia: a 1-year population-based prospective study. Aliment Pharmacol Ther. (2016) 44:592–600. doi: 10.1111/apt.2016.44.issue-6 27444264

[103] Xu Z Ning F Zhang X Wang Q Zhang Y Guo Y . Deciphering the brain-gut axis: elucidating the link between cerebral cortex structures and functional gastrointestinal disorders via integrated Mendelian randomization. Front Neurosci. (2024) 18:1398412/full. doi: 10.3389/fnins.2024.1398412/full 38841096 PMC11152161

[104] Li Z Ma Q Deng Y Rolls ET Shen C Li Y . Irritable bowel syndrome is associated with brain health by neuroimaging, behavioral, biochemical, and genetic analyses. Biol Psychiatry. (2024) 95:1122–32. doi: 10.1016/j.biopsych.2023.12.024 38199582

[105] Sjölund J Kull I Bergström A Ljótsson B Törnblom H Olén O . Quality of life and bidirectional gut-brain interactions in irritable bowel syndrome from adolescence to adulthood. Clin Gastroenterol Hepatol. (2024) 22:858–866.e6. doi: 10.1016/j.cgh.2023.09.022 37802270

[106] Wu MK Huang TL Huang KW Huang YL Hung YY . Association between toll-like receptor 4 expression and symptoms of major depressive disorder. Neuropsychiatr Dis Treat. (2015) 11:1853–7. doi: 10.2147/NDT.S88430 PMC4525784 26257523

[107] Hung YY Kang HY Huang KW Huang TL . Association between toll-like receptors expression and major depressive disorder. Psychiatry Res. (2014) 220:283–6. doi: 10.1016/j.psychres.2014.07.074 25155940

[108] Hung YY Huang KW Kang HY Huang GYL Huang TL . Antidepressants normalize elevated Toll-like receptor profile in major depressive disorder. Psychopharmacol (Berl). (2016) 233:1707–14. doi: 10.1007/s00213-015-4087-7 PMC4828490 26415953

[109] Liu Y Dai C Wang C Wang J Yan W Luo M . Raspberry ketone prevents LPS-induced depression-like behaviors in mice by inhibiting TLR-4/NF-κB signaling pathway via the gut-brain axis. Mol Nutr Food Res. (2024) 68. doi: 10.1002/mnfr.202400090 38757671

[110] Schaper SJ Stengel A . Emotional stress responsivity of patients with IBS-a systematic review. J Psychosom Res. (2022) 153:110694. doi: 10.1016/j.jpsychores.2021.110694 34942583

[111] Fukudo S . Stress and visceral pain: Focusing on irritable bowel syndrome. PAIN. (2013) 154:S63–70. doi: 10.1016/j.pain.2013.09.008 24021863

[112] Taché Y Million M . Role of corticotropin-releasing factor signaling in stress-related alterations of colonic motility and hyperalgesia. J Neurogastroenterol Motil. (2015) 21:8–24. doi: 10.5056/jnm14162 25611064 PMC4288101

[113] Chaniotou Z Giannogonas P Theoharis S Teli T Gay J Savidge T . Corticotropin-releasing factor regulates TLR4 expression in the colon and protects mice from colitis. Gastroenterology. (2010) 139:2083–92. doi: 10.1053/j.gastro.2010.08.024 20732324

[114] Nozu T Miyagishi S Nozu R Takakusaki K Okumura T . Altered colonic sensory and barrier functions by CRF: roles of TLR4 and IL-1. J Endocrinol. (2018) 239:241–52. doi: 10.1530/JOE-18-0441 30139928

[115] Nozu T Miyagishi S Nozu R Takakusaki K Okumura T . Lipopolysaccharide induces visceral hypersensitivity: role of interleukin-1, interleukin-6, and peripheral corticotropin-releasing factor in rats. J Gastroenterol. (2017) 52:72–80. doi: 10.1007/s00535-016-1208-y 27075754

[116] Yuan PQ Wu SV Wang L Tache Y . Corticotropin releasing factor in the rat colon: Expression, localization and upregulation by endotoxin. Peptides. (2010) 31:322–31. doi: 10.1016/j.peptides.2009.11.012 PMC2814976 19944726

[117] Chen H Xu Z Zhao H Cao J Wang R He J . Global research states and trends of micro RNA in irritable bowel syndrome: a bibliometric analysis. Clin Exp Med. (2024) 24:149. doi: 10.1007/s10238-024-01396-y 38967892 PMC11226481

[118] Bravo-Vázquez LA Medina-Ríos I Márquez-Gallardo LD Reyes-Muñoz J Serrano-Cano FI Pathak S . Functional implications and clinical potential of microRNAs in irritable bowel syndrome: A concise review. Dig Dis Sci. (2023) 68:38–53. doi: 10.1007/s10620-022-07516-6 35507132 PMC9066399

[119] Wang W Lou C Gao J Zhang X Du Y . LncRNA SNHG16 reverses the effects of miR-15a/16 on LPS-induced inflammatory pathway. BioMed Pharmacother. (2018) 106:1661–7. doi: 10.1016/j.biopha.2018.07.105 30119242

[120] Yang Y Yang F Yu X Wang B Yang Y Zhou X . miR-16 inhibits NLRP3 inflammasome activation by directly targeting TLR4 in acute lung injury. BioMed Pharmacother. (2019) 112:108664. doi: 10.1016/j.biopha.2019.108664 30784935

[121] Levy RL Jones KR Whitehead WE Feld SI Talley NJ Corey LA . Irritable bowel syndrome in twins: Heredity and social learning both contribute to etiology. Gastroenterology. (2001) 121:799–804. doi: 10.1053/gast.2001.27995 11606493

[122] Camilleri M Carlson P McKinzie S Zucchelli M D’Amato M Busciglio I . Genetic susceptibility to inflammation and colonic transit in lower functional gastrointestinal disorders: preliminary analysis. Neurogastroenterol Motil. (2011) 23:935–E398. doi: 10.1111/j.1365-2982.2011.01749.x 21752155 PMC3173581

[123] Meena NK Verma R Verma N Ahuja V Paul J . TLR4 D299G polymorphism modulates cytokine expression in ulcerative colitis. J Clin Gastroenterol. (2013) 47:773. doi: 10.1097/MCG.0b013e31828a6e93 23470644

[124] Tao E Wu Y Hu C Zhu Z Ye D Long G . Early life stress induces irritable bowel syndrome from childhood to adulthood in mice. Front Microbiol. (2023) 14:1255525. doi: 10.3389/fmicb.2023.1255525 37849921 PMC10577190

[125] Louwies T Mohammadi E Greenwood-Van Meerveld B . Epigenetic mechanisms underlying stress-induced visceral pain: Resilience versus vulnerability in a two-hit model of early life stress and chronic adult stress. Neurogastroenterol Motil. (2023) 35:e14558. doi: 10.1111/nmo.14558 36893055

[126] Rawat K Singh N Kumari P Saha L . A review on preventive role of ketogenic diet (KD) in CNS disorders from the gut microbiota perspective. Rev Neurosci. (2021) 32:143–57. doi: 10.1515/revneuro-2020-0078 33070123

[127] Brietzke E Mansur RB Subramaniapillai M Balanzá-Martínez V Vinberg M González-Pinto A . Ketogenic diet as a metabolic therapy for mood disorders: Evidence and developments. Neurosci Biobehav Rev. (2018) 94:11–6. doi: 10.1016/j.neubiorev.2018.07.020 30075165

[128] Dai YQ Weng H Wang Q Guo XJ Wu Q Zhou L . Moxibustion for diarrhea-predominant irritable bowel syndrome: A systematic review and meta-analysis of randomized controlled trials. Complement Ther Clin Pract. (2022) 46:101532. doi: 10.1016/j.ctcp.2021.101532 35051805

[129] Wu IXY Wong CHL Ho RST Cheung WKW Ford AC Wu JCY . Acupuncture and related therapies for treating irritable bowel syndrome: overview of systematic reviews and network meta-analysis. Ther Adv Gastroenterol. (2019) 12. doi: 10.1177/1756284818820438 PMC6348567 30719074

[130] Zheng H Jin S Shen YL Peng WY Ye K Tang TC . Chinese herbal medicine for irritable bowel syndrome: A meta-analysis and trial sequential analysis of randomized controlled trials. Front Pharmacol. (2021) 12:694741. doi: 10.3389/fphar.2021.694741 34385918 PMC8353248

[131] Wu YB Dai YK Zhang L Pan HG Chen WJ Li RL . Pharmacological treatments of Chinese herbal medicine for irritable bowel syndrome in adults: A network meta-analysis of randomized controlled trials. PloS One. (2021) 16:e0255665. doi: 10.1371/journal.pone.0255665 34358263 PMC8345858

[132] Hawrelak JA Wohlmuth H Ptatinson M Myers SP Goldenberg JZ Harnett J . Western herbal medicines in the treatment of irritable bowel syndrome: A systematic review and meta-analysis. Complement Ther Med. (2020) 48:102233. doi: 10.1016/j.ctim.2019.102233 31987249

[133] Esmaealzadeh N Ram M Abdolghaffari A Marques AM Bahramsoltani R . Toll-like receptors in inflammatory bowel disease: A review of the role of phytochemicals. Phytomedicine. (2024) 123:155178. doi: 10.1016/j.phymed.2023.155178 38007993

[134] Zulkefli N Zahari CNMC Sayuti NH Kamarudin AA Saad N Hamezah HS . Flavonoids as potential wound-healing molecules: emphasis on pathways perspective. Int J Mol Sci. (2023) 24:4607. doi: 10.3390/ijms24054607 36902038 PMC10003005

[135] Xiong HH Lin SY Chen LL Ouyang KH Wang WJ . The interaction between flavonoids and intestinal microbes: A review. FOODS. (2023) 12:320. doi: 10.3390/foods12020320 36673411 PMC9857828

[136] Pimentel M Lembo A . Microbiome and its role in irritable bowel syndrome. Dig Dis Sci. (2020) 65:829–39. doi: 10.1007/s10620-020-06109-5 32026278

[137] So D Quigley EMM Whelan K . Probiotics in irritable bowel syndrome and inflammatory bowel disease: review of mechanisms and effectiveness. Curr Opin Gastroenterol. (2023) 39:103. doi: 10.1097/MOG.0000000000000902 36821458

